# Phenotypic and molecular characterization of antimicrobial resistance in *Enterobacter* spp. isolates from companion animals in Japan

**DOI:** 10.1371/journal.pone.0174178

**Published:** 2017-03-22

**Authors:** Kazuki Harada, Takae Shimizu, Yujiro Mukai, Ken Kuwajima, Tomomi Sato, Akari Kajino, Masaru Usui, Yutaka Tamura, Yui Kimura, Tadashi Miyamoto, Yuzo Tsuyuki, Asami Ohki, Yasushi Kataoka

**Affiliations:** 1 Department of Veterinary Internal Medicine, Tottori University, Tottori, Japan; 2 Laboratory of Veterinary Microbiology, Nippon Veterinary and Life Science University, Tokyo, Japan; 3 Laboratory of Food Microbiology and Food Safety, Rakuno Gakuen University, Hokkaido, Japan; 4 Miyamoto Animal Hospital, Yamaguchi, Japan; 5 Sanritsu Zelkova Veterinary Laboratory, Kanagawa, Japan; 6 Fujifilm Monoris Co., Ltd., Tokyo, Japan; Ross University School of Veterinary Medicine, SAINT KITTS AND NEVIS

## Abstract

The emergence of antimicrobial resistance among *Enterobacter* spp., including resistance to extended-spectrum cephalosporins (ESC), is of great concern in both human and veterinary medicine. In this study, we investigated the prevalence of antimicrobial resistance among 60 isolates of *Enterobacter* spp., including *E*. *cloacae* (n = 44), *E*. *aerogenes* (n = 10), and *E*. *asburiae* (n = 6), from clinical specimens of dogs and cats from 15 prefectures in Japan. Furthermore, we characterized the resistance mechanisms harbored by these isolates, including extended-spectrum β-lactamases (ESBLs) and plasmid-mediated quinolone resistance (PMQR); and assessed the genetic relatedness of ESC-resistant *Enterobacter* spp. strains by multilocus sequence typing (MLST) and pulsed-field gel electrophoresis (PFGE). Antimicrobial susceptibility testing demonstrated the resistance rates to ampicillin (93.3%), amoxicillin-clavulanic acid (93.3%), cefmetazole (93.3%), chloramphenicol (46.7%), ciprofloxacin (43.3%), tetracycline (40.0%), ceftazidime (33.3%), cefotaxime (33.3%), trimethoprim/sulfamethoxazole (28.3%), gentamicin (23.3%), and meropenem (0%). Phenotypic testing detected ESBLs in 16 of 18 ESC-resistant *E*. *cloacae* isolates but not in the other species. The most frequent ESBL was CTX-M-15 (n = 8), followed by SHV-12 (n = 7), and CTX-M-3 (n = 1). As for AmpC β-lactamases, CMY-2 (n = 2) and DHA-1 (n = 2) were identified in ESC-resistant *E*. *cloacae* strains with or without ESBLs. All of the ESC-resistant *E*. *cloacae* strains also harbored one or two PMQRs, including *qnrB* (n = 15), *aac(6’)-Ib-cr* (n = 8), and *qnrS* (n = 2). Based on MLST and PFGE analysis, *E*. *cloacae* clones of ST591-SHV-12, ST171-CTX-M-15, and ST121-CTX-M-15 were detected in one or several hospitals. These results suggested intra- and inter-hospital dissemination of *E*. *cloacae* clones co-harboring ESBLs and PMQRs among companion animals. This is the first report on the large-scale monitoring of antimicrobial-resistant isolates of *Enterobacter* spp. from companion animals in Japan.

## Introduction

Members of the genus *Enterobacter*, belonging to the *Enterobacteriaceae*, are Gram-negative bacilli that inhabit terrestrial and aquatic environments including water, sewage, and soil, as well as the intestinal tracts of mammals [1]. *Enterobacter cloacae* is the most medically-important species in the genus and is responsible for nosocomial infections in humans [2]. In companion animals, this bacterial species is rarely associated with urinary tract infections, wound infections, pneumonia, intravenous catheter site infections, otitis externa, peritonitis, and dermatitis [3].

The emergence of antimicrobial resistance among *Enterobacter* spp. is of great concern worldwide in human medicine [1,2]. It increases the risk of antimicrobial treatment failure not only in humans but also in companion animals. Similarly, the emergence of antimicrobial-resistant bacteria in companion animals may have important human public health consequences if isolates are transmitted to humans from their pets [4,5]. Understanding the prevalence of antimicrobial resistance among *Enterobacter* spp. isolates is thus important both from veterinary medicine and public health perspectives.

Resistance to extended-spectrum cephalosporins (ESC) among Gram-negative bacteria, including *Enterobacter* spp., is of particular concern [6]. In *Enterobacter* spp., ESC resistance is most typically caused by the overproduction of AmpC β-lactamases, which is due to the derepression of a chromosomal gene or the acquisition of a transferable AmpC β-lactamase [1,6]. In addition, extended-spectrum β-lactamases (ESBLs) and carbapenemases have been identified in *Enterobacter* spp. [7], exacerbating the issue of ESC resistance. An even greater concern is that most ESC-resistant *Enterobacteriaceae* exhibit multidrug resistance, including fluoroquinolone resistance, mainly due to chromosomal mutations in the enzymes targeted by the drug, and plasmid-mediated quinolone resistance (PMQR) [8]. In recent years, these resistance mechanisms have been well documented among *Enterobacter* spp. isolates from companion animals across several countries, including Australia [9,10], France [11], and Germany [12]. However, the status of emerging antimicrobial resistance among *Enterobacter* spp. in companion animals remains unknown in many other countries, including Japan.

The aim of the present study was to investigate the prevalence of antimicrobial resistance, and provide molecular characterization of ESC resistance and PMQR among *Enterobacter* spp. isolates from clinical specimens taken from dogs and cats that visited veterinary hospitals throughout Japan. A further aim was to assess the epidemiological relatedness of ESC-resistant *Enterobacter* spp. strains.

## Materials and methods

### Bacterial isolates

A total of 60 clinical isolates of *Enterobacter* spp., consisting of *E*. *cloacae* (n = 44), *E*. *aerogenes* (n = 10), and *E*. *asburiae* (n = 6), were collected from dogs (n = 44) and cats (n = 16) kept by different owners that visited veterinary hospitals between 2003 and 2015. These hospitals were located at the following 15 prefectures in Japan: Hokkaido, Fukui, Gunma, Ibaraki, Saitama, Tokyo, Chiba, Kanagawa, Nagano, Aichi, Osaka, Hyogo, Tottori, Yamaguchi, and Fukuoka prefectures. The specimens were isolated from various anatomical sites, assessed as being sites of bacterial infection by clinical veterinarians, including the urinary tract (n = 27), pus from unspecified locations (n = 10), nasal cavity (n = 5), ear (n = 4), skin (n = 2), eye (n = 2), ascites (n = 2), and the other sites (n = 8). The details of *Enterobacter* spp. isolates used in this study are shown in S1 Table. No information was available regarding previous antimicrobial treatment of the dogs and cats. Ethical approval was not needed according to the ethical guidelines for epidemiological research by the Japanese government because this study focused on bacterial aspects. Bacterial identification was conducted by assessing the growth status on CHROMagar orientation medium [13], using the API 20E kit (SYSMEX bioMérieux Co., Ltd., Tokyo, Japan), and MALDI-TOF MS with the Bruker MALDI Biotyper system (Bruker Daltonics, Bremen, Germany) [14]. All confirmed *Enterobacter* spp. isolates were stored at −80°C in 10% skim milk.

### Antimicrobial susceptibility testing

Susceptibilities to ampicillin (AMP, Wako Pure Chemical Industries, Ltd., Osaka, Japan), amoxicillin-clavulanic acid (ACV, Sigma-Aldrich Co. LLC., Tokyo, Japan), cefmetazole (CMZ, Sigma-Aldrich), cefotaxime (CTX, Wako Pure Chemical), ceftazidime (CAZ, Sigma-Aldrich), meropenem (MPM, Wako Pure Chemical), tetracycline (TET, Wako Pure Chemical), gentamicin (GEN, Sigma-Aldrich), chloramphenicol (CHL, Wako Pure Chemical), trimethoprim/sulfamethoxazole (TMS, Wako Pure Chemical), and ciprofloxacin (CIP, Wako Pure Chemical) were determined. Susceptibility testing was conducted using the agar dilution method, according to the Clinical and Laboratory Standards Institute (CLSI) guidelines [15]. The results obtained were interpreted according to the criteria contained within the CLSI guidelines [16,17]. *Escherichia coli* ATCC 25922 was used as a control strain.

### Phenotypic analysis of ESC-resistant *Enterobacter* spp. strains

ESC-resistant [i.e. minimum inhibitory concentration (MIC) for CTX or CAZ of ≥ 2 or 8 μg/mL, respectively] strains were screened for ESBLs by the double-disc synergy test using CTX, CAZ, cefepime, and ACV disks on Mueller-Hinton agar plates without or with 200 μg/mL cloxacillin [18]. In addition, ESC-resistant isolates without synergism with clavulanate and with inhibition zones augmented upon cloxacillin were classified as organisms overexpressing AmpC β-lactamase [19].

Isolates with an MIC for MPM of ≥ 0.25 μg/mL, the recommended cut-off value for carbapenemase-producing *Enterobacteriaceae* by the European Committee on Antimicrobial Susceptibility Testing [20], were screened for the production of *Klebsiella pneumoniae* carbapenemase (KPC) and metallo-β-lactamase, and porin loss, using a D70C carbapenemase detection set (MASTDISCS^™^ID, UK).

### Characterization of β-lactamase genes and PMQR in ESC-resistant *Enterobacter* spp. isolates

Genomic DNA from each of the isolates was prepared by suspending several colonies in 0.5 mL of water and boiling for 10 min. These samples were used as templates for further genetic analyses. All of the ESC-resistant strains were screened for class A β-lactamase genes (i.e. *bla*TEM and *bla*SHV), which were identified using PCR and DNA sequencing, as previously reported [21]. Class D β-lactamase genes (*bla*OXA) were detected using multiplex PCR [22], and were amplified and bi-directionally sequenced using specific primers [23]. In addition, AmpC β-lactamase genes (i.e. the ACC, FOX, MOX, DHA, CIT, and EBC groups) were screened by multiplex PCR [24], and were amplified and then bi-directionally sequenced using specific primers [25,26]. In ESBL-positive strains, the CTX-M-type β-lactamase genes were detected using multiplex PCR [27]; for the positive isolates, the genes were amplified and sequenced to identify CTX-M subtypes using group-specific PCR primers [21].

All ESC-resistant isolates were screened for eight PMQR genes (i.e. *qnrA*, *qnrB*, *qnrC*, *qnrD*, *qnrS*, *qepA*, *aac(6’)-Ib-cr*, and *oqxAB*) using multiplex PCR [28]. Positive results were confirmed by individual gene PCRs. Randomly selected PCR products of PMQR genes were directionally sequenced with the same primers for confirmation.

### Multilocus sequence typing and pulsed-field gel electrophoresis of ESC-resistant *E*. *cloacae* strains

For ESC-resistant *E*. *cloacae* strains, multilocus sequence typing (MLST) with seven genes (i.e. *dnaA*, *fusA*, *gyrB*, *leuS*, *pyrG*, *rplB*, and *rpoB*) was carried out as described previously [29]. A new sequence type (ST) was submitted to the MLST website and new ST numbers were assigned. The eBURST v3 analysis (http://eburst.mlst.net/v3/instructions/) was performed to assess the relatedness between STs.

Pulsed-field gel electrophoresis (PFGE) was performed on ESC-resistant *E*. *cloacae* strains, as previously described [30]. DNA embedded in agarose was digested with *Xba*I (Takara Bio, Inc., Tokyo, Japan) and then electrophoresed using CHEF DRIII (Bio-Rad, Hercules, CA, USA). PFGE profiles were digitized for analysis using BioNumerics software (version 5.10; Applied Maths, TX, USA). All fragment sizes within the gel were normalized using the molecular weight method. A similarity matrix was calculated using the Dice coefficient, and cluster analysis was performed using the UPGMA algorithm. A cluster was defined based on a similarity cut-off of 80% with 1.0% optimization and 1.0% band tolerance.

## Results

### Rates of antimicrobial resistance among *Enterobacter* spp. isolates

**Fig 1:**
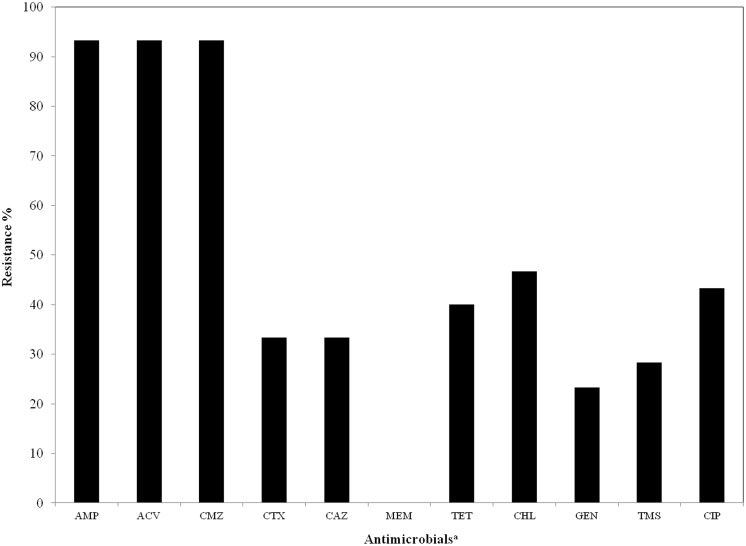
Rates of resistance to 11 antimicrobials among *Enterobacter* isolates (n = 60) from companion animals. ^a^AMP, ampicillin; ACV, amoxicillin-clavulanic acid; CMZ, cefmetazole; CTX, cefotaxime; CAZ, ceftazidime, MPM, meropenem; TET, tetracycline; GEN, gentamicin; CHL, chloramphenicol; TMS, trimethoprim/sulfamethoxazole; CIP, ciprofloxacin.

The numbers of isolates with resistance to AMP, ACV, CMZ, CHL, CIP, TET, CAZ, CTX, TMS, GEN, and MPM were 56 (93.3%), 56 (93.3%), 56 (93.3%), 28 (46.7%), 26 (43.3%), 24 (40.0%), 20 (33.3%), 20 (33.3%), 17 (28.3%), 14 (23.3%), and 0 (0%), respectively, in 60 *Enterobacter* spp. clinical isolates (Fig 1).

### Identification of β-lactamases and PMQR genes in ESC-resistant *Enterobacter* spp. strains

Resistance to ESC (CTX and CAZ) was detected in 20 isolates, consisting of *E*. *cloacae* (n = 18) and *E*. *asburiae* (n = 2). The double-disc synergy test revealed that 16 of 18 ESC-resistant *E*. *cloacae* strains produced ESBLs, but no *E*. *asburiae* strains displayed this phenotype. Four non-ESBL-producing ESC-resistant strains were phenotypically identified as AmpC hyperproducers. In total, 16 of 60 (26.7%) *Enterobacter* spp. isolates were positive for ESBLs.

Table 1 shows the detailed characteristics of 20 ESC-resistant *Enterobacter* spp. strains. The CTX-M-15 (n = 8), SHV-12 (n = 7), and CTX-M-3 (n = 1) were detected in ESBL-producing *E*. *cloacae* strains. As for AmpC β-lactamases, CMY-2 and DHA-1 were detected together with/without ESBLs in two ESC-resistant *E*. *cloacae* strains each, whereas ACT-type genes were detected in two ESC-resistant *E*. *asburiae* strains. TEM-1 and OXA-1 were also detected in 13 and 7 ESC-resistant *E*. *cloacae* strains, respectively. Seven of the ESC-resistant strains had higher MPM MICs (0.25–4 μg/mL) than the screening cut-offs for carbapenemase-positive *Enterobacteriaceae* [18]. The results of the disk test indicated that all of the seven strains have porin loss, in addition to ESBLs, but not have carbapenemases. Of the eight PMQR genes tested, *qnrB*, *aac(6’)-Ib-cr*, and *qnrS* were detected in 15, 8, and 2 ESC-resistant isolates, respectively.

**Table 1:** Characterization of 20 ESC-resistant *Enterobacter* spp. strains from dogs and cats in Japan.

Strain	Host	ST	AmpC overexpression	ESBL/AmpC	Other β-lactamase	PMQR	MIC(μg/mL)^b^									
							ACV	CMZ	CTX	CAZ	MPM	TET	CHL	GEN	TMS	CIP
*E*. *cloacae* (n = 18)																
EN13	Dog	113	+	Not detected	TEM-1	*qnrS*	64/32	256	64	32	0.125	256	>256	0.5	>64/1216	16
EN41	Dog	114	-	CTX-M-15	OXA-1	*aac(6’)-Ib-cr*, *qnrB*	64/32	>256	256	32	0.06	8	32	32	>64/1216	128
EN33	Dog	121	-	CTX-M-15	TEM-1, OXA-1	*aac(6’)-Ib-cr*, *qnrB*	64/32	128	>256	64	0.125	64	256	16	>64/1216	64
EN72	Dog	121	-	CTX-M-15	OXA-1	*aac(6’)-Ib-cr*, *qnrB*	128/64	256	>256	32	0.03	32	>256	16	>64/1216	>32
EN73	Dog	121	-	CTX-M-15	OXA-1	*aac(6’)-Ib-cr*, *qnrB*	64/32	256	>256	32	0.03	32	>256	16	>64/1216	>32
EN28	Dog	136	-	CTX-M-15, CMY-2	TEM-1, OXA-1	*aac(6’)-Ib-cr*, *qnrB*	64/32	256	>256	256	0.06	64	256	0.5	>64/1216	128
EN59	Dog	171	-	CTX-M-15	TEM-1, OXA-1	*aac(6’)-Ib-cr*	64/32	64	>256	32	0.06	32	256	1	>64/1216	8
EN63	Dog	171	-	CTX-M-15, DHA-1	Not detected	*aac(6’)-Ib-cr*, *qnrB*	64/32	128	256	64	0.03	64	256	128	>64/1216	64
EN66	Dog	171	-	CTX-M-15	TEM-1, OXA-1	*aac(6’)-Ib-cr*, *qnrB*	64/32	64	>256	128	0.125	32	256	32	>64/1216	64
EN60	Dog	544	+	DHA-1	Not detected	*qnrB*	64/32	256	32	32	0.06	8	32	128	1/19	2
EN3	Dog	591	-	SHV-12	TEM-1	*qnrB*	64/32	>256	64	128	0.25	256	>256	256	0.5/9.5	64
EN4	Dog	591	-	SHV-12	TEM-1	*qnrB*	64/32	>256	64	128	0.25	256	>256	128	0.5/9.5	64
EN5	Dog	591	-	SHV-12	TEM-1	*qnrB*	64/32	>256	64	256	2	256	>256	256	0.5/9.5	64
EN7	Cat	591	-	SHV-12, CMY-2	TEM-1	*qnrB*	128/64	>256	256	256	4	256	>256	128	1/19	64
EN10	Dog	591	-	SHV-12	TEM-1	*qnrB*	64/32	>256	128	128	1	>256	>256	64	1/19	64
EN12	Dog	591	-	SHV-12	TEM-1	*qnrB*	64/32	>256	128	256	1	256	>256	64	1/19	64
EN14	Cat	591	-	SHV-12	TEM-1	*qnrB*	64/32	>256	32	128	0.25	256	>256	128	1/19	128
EN53	Cat	813^a^	-	CTX-M-3	TEM-1	*qnrS*	64/32	128	256	4	0.06	256	>256	128	>64/1216	0.5
*E*. *asburiae* (n = 2)																
EN6	Dog	-	+	ACT-8	Not detected	Not detected	128/64	>256	16	32	0.03	1	8	0.5	0.125/2.375	4
EN20	Dog	-	+	ACT-3	Not detected	Not detected	64/32	256	64	64	0.125	2	32	2	2/38	16

^a^ New ST.

^b^ ACV, amoxicillin-clavulanic acid; CMZ, cefmetazole; CTX, cefotaxime; MPM, meropenem; TET, tetracycline; GEN, gentamicin; CHL, chloramphenicol; TMS, trimethoprim/sulfamethoxazole; CIP, ciprofloxacin. The MIC values of ampicillin were >256 μg/mL in all ESC-resistant *Enterobacter* spp. strains.

### MLST typing of ESC-resistant *Enterobacter* spp. strains

**Fig 2:**
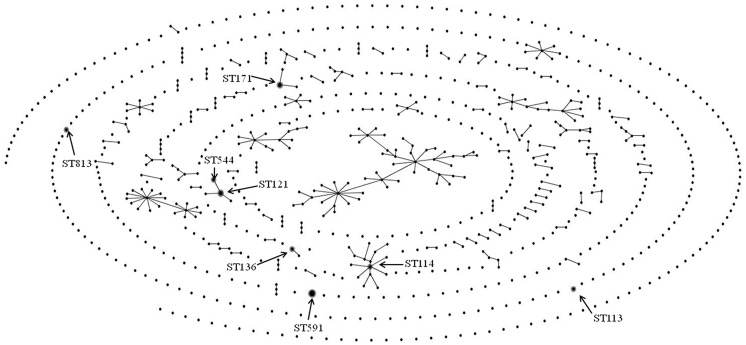
Population snapshot by eBURST analysis of ESC-resistant *E*. *cloacae* strains against the entire *E*. *cloacae* MLST database. *The STs identified in this study are labeled with arrows. The names of the clonal complexes are based on the ST assigned as the founder genotype. The relative size of the circles indicates the prevalence of STs and lines between STs connect single locus variants.

As shown in Table 1, 18 ESC-resistant *E*. *cloacae* isolates investigated by MLST were assigned to eight STs: ST591 (allelic profile 3-3-110-232-19-16-17, n = 7), ST121 (10-21-9-44-45-4-32, n = 3), ST171 (49-21-19-44-45-12-32, n = 3), ST113 (4-22-68-69-37-4-24, n = 1), ST114 (53-35-20-44-45-4-6, n = 1), ST136 (74-21-74-44-45-4-6, n = 1), ST544 (10-21-9-44-45-4-33, n = 1), and ST813 (74-20-20-44-99-24-32, n = 1). Fig 2 illustrates a population snapshot by eBURST analysis of our collection of isolates, against 827 previously-reported STs obtained from the MLST database (accessed on 30 July 2016). Of the eight STs, ST121 and ST544 were included in the same clonal complex, whereas the remaining STs were singletons or had single locus variants that were unrelated to the STs in our collection.

### PFGE analysis of ESC-resistant *Enterobacter* spp. strains

**Fig 3:**
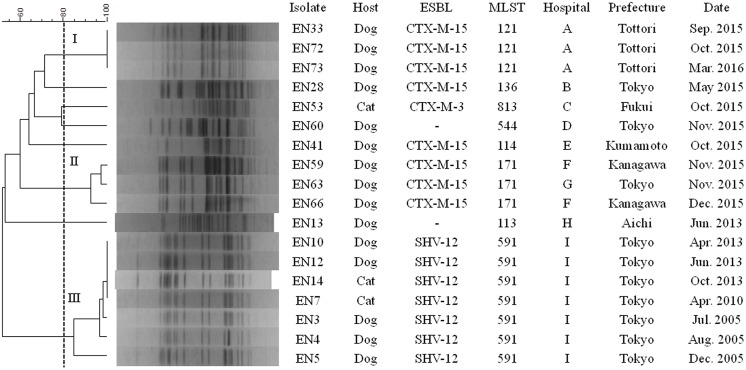
PFGE profiles of 18 ESC-resistant *E*. *cloacae* strains from companion animals in Japan. *The numbers embedded in the phylogenetic tree indicate clusters.

In PFGE analysis, ESC-resistant *E*. *cloacae* strains formed three distinct clusters (Fig 3). Clusters I and III consisted of three ST121-CTX-M-15 strains and seven ST591-SHV-12 strains, respectively, obtained from the same veterinary hospital. In addition, cluster II contained two ST171-CTX-M-15 strains obtained from the same hospital.

## Discussion

There have been few reports on the prevalence of antimicrobial resistance in overall populations of *Enterobacter* spp. isolates from companion animals worldwide. This study demonstrated that almost all *Enterobacter* spp. isolates in our collection exhibited resistance to β-lactams excluding AMP, ACV, and CMZ, possibly due to chromosomal AmpC β-lactamases [1,2]. The rates of resistance to CTX and CIP in our collection (33.3% and 43.3%, respectively) were similar to those in *K*. *pneumoniae* isolates (39.3% and 41.6%, respectively) [31], but were much higher than those in *Proteus mirabilis* isolates (1.9% and 5.8%, respectively) [32] from companion animals in Japan. Compared with *Enterobacter* spp. isolates from human in Japan [33], the rate of CIP resistance in our collection was extremely high (4.4% vs. 43.3%). Similarly, a higher rate of CIP resistance than human isolates has been reported in *E*. *coli* [34] and *K*. *pneumoniae* [31] isolates from companion animals. These findings implied the heavy use of fluoroquinolone drugs in veterinary medicine. To evaluate inter-country diversity of the prevalence of antimicrobial-resistant *Enterobacter* spp. from companion animals, systematic surveillance would be needed in many countries.

We found higher prevalence of ESBLs in *Enterobacter* isolates (16/60, 26.7%), compared with that reported from dogs (11/314, 3.5%) and cats (11/108, 10.2%) in France [11] and human isolates in Japan (22/364, 6.0%) [35]. In addition, the carriage rate of ESBLs in our collection was slightly lower that in *K*. *pneumoniae* isolates (31/89, 34.8%) [31], but was much higher than that in *P*. *mirabilis* isolates (0/103, 0%) [32] from companion animals in Japan. These data suggest that the risk of ESBL carriage is relatively high in *Enterobacter* spp. isolates from companion animals in Japan. In this study, the major types of ESBLs were CTX-M-15 and SHV-12. Similarly, these ESBLs have been frequently detected in companion animals in France and Australia [9,11]. As for ESBLs detected in human strains in Japan, CTX-M-3, which was an uncommon ESBL in this study (n = 1), was predominant, whereas CTX-M-15 was not detected [35]. These data suggest that the major types of ESBLs differ between *Enterobacter* spp. isolates from companion animals and human. Therefore, the risk of zoonotic transmission of ESBL-producing *Enterobacter* spp. is not likely to be high, although attention should be paid to *Enterobacter* spp. isolates from companion animals as reservoirs of ESBLs.

Besides ESBLs, we identified several AmpC β-lactamase genes (ACT, CMY, and DHA-type genes) in ESC-resistant strains. Notably, ACT-type enzymes are known to be representative chromosomal AmpC β-lactamases of *Enterobacter* spp. [36]. As for ACT-type genes, two *E*. *asburiae* strains were positive, but all of the *E*. *cloacae* strains were negative, which might imply the lack of AmpC genes or sequence variability among chromosomal AmpC genes, as previously reported [37]. The remaining two types of plasmid-mediated AmpC β-lactamases have rarely been identified in *Enterobacter* spp. isolated from humans [38,39]. We also found that several non-ESBL-producing ESC-resistant strains overexpressed chromosomal AmpC β-lactamase. Our findings suggest that plasmid-mediated AmpC β-lactamases, in addition to chromosomal AmpC β-lactamases, partly contribute to ESC resistance in *Enterobacter* spp. isolates from companion animals. On the other hand, all ESC-resistant *Enterobacter* spp. strains were negative for carbapenemases. This finding indicates that carbapenemases are uncommon among *Enterobacter* spp. isolates from companion animals in Japan, as well as among other Gram-negative bacteria [31,32,40,41], although they have been reported among animal isolates in other countries [12] and among human isolates in Japan [42].

All of the ESC-resistant *E*. *cloacae* strains possessed one or more PMQR genes, which is likely to contribute to the extremely high rate of CIP resistance among these strains (18/20, 90.0%). We found the high prevalence of *qnrB* and *aac(6’)-Ib-cr* among ESC-resistant *Enterobacter* strains, and similar findings have been confirmed among *Enterobacter* isolates from companion animals in Australia [10]. Notably, most *aac(6’)-Ib-cr*-positive strains also harbored OXA-1 and/or CTX-M-15 β-lactamases, which may indicate a strong association between these genes [43,44]. In addition, we detected the *qnrS* gene, which is the most prevalent PMQR gene among ESBL-producing *Enterobacter* spp. isolates from humans in Japan [35] but was not previously detected among isolates from companion animals in Australia [10]. These findings may suggest that *qnrS* gene is locally spread among companion animals and humans in Japan.

Recently, Izdebski *et al*. [19] found that ST66, ST78, ST108, and ST114 strains were widely spread as high-risk international clones of ESC-resistant *E*. *cloacae*. Of these STs, ST114 was identified in our collection and was associated with CTX-M-15. This is the first report of the ST114-CTX-M-15 clone in companion animals in any country other than France [11]. Furthermore, we found eight STs that had not been identified in the study by Haenni *et al*. [11]. This implies that ESC-resistant *E*. *cloacae* isolated from different countries generally belong to different lineages. Unfortunately, the prevalence of STs has not been reported for human ESC-resistant *E*. *cloacae* isolates in Japan, and thus further studies to compare STs between animal and human isolates are needed.

Based on the PFGE analysis, we found that ESBL-producing *E*. *cloacae* clones were disseminated among different patients in the same hospital. This result strongly suggests nosocomial infections of ESBL-producing *E*. *cloacae* clones, and similar findings have previously been reported [11]. Surprisingly, several clones were repeatedly identified at an interval of many months, implying that ESBL-producing *E*. *cloacae* clones can survive inside or outside of hospitals for a long period [45]. Therefore, the dissemination of ESBL-producing *E*. *cloacae* clones among companion animals may occur not only via direct spread from animal to animal, but also via indirect transmission from potential reservoirs and sources in the environment. Our data emphasize the need for infection control in hospitals and in the community to prevent dissemination of ESBL-producing *E*. *cloacae* clones among companion animals.

## Conclusion

Our data demonstrate the high prevalence of ESBLs and PMQR genes among ESC-resistant *E*. *cloacae* strains isolated from companion animals in Japan. Epidemiological data suggest that *E*. *cloacae* clones co-harboring ESBLs and PMQR genes are disseminated via intra-hospital and inter-hospital transmission.

## Supporting information

S1 Table The details of *Enterobacter* spp. isolates used in this study. *AMP, ampicillin; ACV, amoxicillin-clavulanic acid; CMZ, cefmetazole; CTX, cefotaxime; CAZ, ceftazidime, MPM, meropenem; TET, tetracycline; GEN, gentamicin; CHL, chloramphenicol; TMS, trimethoprim/sulfamethoxazole; CIP, ciprofloxacin; R, resistant.
(XLS)

## References

[1] Davin-Regli A, Pagès JM. *Enterobacter aerogenes* and *Enterobacter cloacae*; versatile bacterial pathogens confronting antibiotic treatment. Front Microbiol. 2015; 6: Article 392 10.3389/fmicb.2015.00392 26042091 PMC4435039

[2] Mezzatesta ML, Gona F, Stefani S. *Enterobacter cloacae* complex: clinical impact and emerging antibiotic resistance. Future Microbiol. 2012; 7: 887–902. 10.2217/fmb.12.61 22827309

[3] Weese JS. Investigation of *Enterobacter cloacae* infections at a small animal veterinary teaching hospital. Vet Microbiol. 2008; 130: 426–428. 10.1016/j.vetmic.2008.02.009 18374521

[4] Guardabassi L, Schwarz S, Lloyd DH. Pet animals as reservoirs of antimicrobial-resistant bacteria. J Antimicrob Chemother. 2004; 54: 321–332. 10.1093/jac/dkh332 15254022

[5] Lloyd DH. Reservoirs of antimicrobial resistance in pet animals. Clin Infect Dis. 2007; 45: S148–152. 10.1086/519254 17683019

[6] Paterson DL. Resistance in gram-negative bacteria: *Enterobacteriaceae*. Am J Infect Control 2006; 34: S20–28. 10.1016/j.ajic.2006.05.238 16813978

[7] Bush K. Alarming β-lactamase-mediated resistance in multidrug-resistant *Enterobateriaceae*. Curr Opin Microbiol. 2010; 13: 558–564. 10.1016/j.mib.2010.09.006 20920882

[8] Delgado-Valverde M, Sojo-Dorado J, Pascual A, Rodríguez-Baňo J. Clinical management of infections caused by multidrug-resistant *Enterobacteriaceae*. Ther Adv Infect Dis. 2013; 1: 49–69. 10.1177/2049936113476284 25165544 PMC4040721

[9] Sidjabat HE, Hanson ND, Smith-Moland E, Bell JM, Gibson JS, Filippich LJ, et al Identification of plasmid-mediated extended-spectrum and AmpC β-lactamases in *Enterobacter* spp. isolated from dogs. J Med Microbiol. 2007; 56: 426–434. 10.1099/jmm.0.46888-0 17314376

[10] Gibson JS, Cobbold RN, Heisig P, Sidjabat HE, Kyaw-Tanner MT, Trott DJ. Identification of Qnr and AAC(6’)-1b-cr plasmid-mediated fluoroquinolone resistance determinants in multidrug-resistant Enterobacter spp. isolated from extraintestinal infections in companion animals. Vet Microbiol. 2010; 143: 329–336. 10.1016/j.vetmic.2009.11.031 20036084

[11] Haenni M, Saras E, Ponsin C, Dahmen S, Petitjean M, Hocquet D, et al High prevalence of international ESBL CTX-M-15-producing *Enterobacter cloacae* ST114 clone in animals. J Antimicrob Chemother. 2016; 71: 1497–1500. 10.1093/jac/dkw006 26850718

[12] Schmiedel J, Falgenhauer L, Domann E, Bauerfeind R, Prenger-Berninghoff E, Imirzalioglu C, et al Multiresistant extended-spectrum β-lactamase-producing *Enterobacteriaceae* from humans, companion animals and horses in central Hesse, Germany. BMC Microbiol. 2014; 14: 187 10.1186/1471-2180-14-187 25014994 PMC4105247

[13] Ohkusu K. Cost-effective and rapid presumptive identification of gram-negative bacilli in routine urine, pus and stool culture: Evaluation of the use of CHROMagar orientation medium in conjunction with biochemical tests. J Clin Microbiol. 2000; 38: 4586–4592. 11101600 10.1128/jcm.38.12.4586-4592.2000 PMC87641

[14] Dierig A, Frei R, Egli A. The fast route to microbe identification: matrix assisted laser desorption/ionization-time of flight mass spectrometry (MALDI-TOF-MS). Pediatr Infect Dis J. 2015; 34: 97–99. 10.1097/INF.0000000000000601 25741802

[15] Clinical and Laboratory Standards Institute. Performance Standards for Antimicrobial Disk and Dilution Susceptibility Tests for Bacteria Isolated From Animals. Approved Standard-Fourth Edition. CLSI document VET01-A4, Wayne, PA., USA. 2013.

[16] Clinical and Laboratory Standards Institute. Performance Standards for Antimicrobial Disk and Dilution Susceptibility Tests for Bacteria Isolated From Animals. Second Informational Supplement. CLSI document VET01-S2, Wayne, PA., USA. 2013.

[17] Clinical and Laboratory Standards Institute. Performance Standards for Antimicrobial Susceptibility Testing; Twntieth Informational Supplement. CLSI document M100-S20, Wayne, PA., USA. 2010.

[18] EUCAST. EUCAST Guidelines for Detection of Resistance Mechanisms and Specific Resistances of Clinical and/or Epidemiological Importance. 2013. http://www.eucast.org/fileadmin/src/media/PDFs/EUCAST_files/Resistance_mechanisms/EUCAST_detection_of_resistance_mechanisms_v1.0_20131211.pdf.

[19] Izdebski R, Baraniak A, Herda M, Fiett J, Bonten MJ, Carmeli Y, et al MLST reveals potentially high-risk international clones of *Enterobacter cloacae*. J Antimicrob Chemother. 2015; 70: 48–56. 10.1093/jac/dku359 25216820

[20] Hrabák J, Chudáčková E, Papagiannitsis CC. Detection of carbapenemases in *Enterobacteriaceae*: a challenge for diagnostic microbiological laboratories. Clin Microbiol Infect. 2014; 20: 839–853. 10.1111/1469-0691.12678 24813781

[21] Kojima A, Ishii Y, Ishihara K, Esaki H, Asai T, Oda C, et al Extended-spectrum-β-lactamase-producing *Escherichia coli* strains isolated from farm animals from 1999 to 2002: report from the Japanese Veterinary Antimicrobial Resistance Monitoring Program. Antimicrob Agents Chemother. 2005; 49: 3533–3537. 10.1128/AAC.49.8.3533-3537.2005 16048977 PMC1196269

[22] Voets GM, Fluit AC, Scharringa J, Cohen Stuart J, Leverstein-van Hall MA. A set of multiplex PCRs for genotypic detection of extended-spectrum β-lactamases, carbapenemases, plasmid-mediated AmpC β-lactamases and OXA β-lactamases. Int J Antimicrob Agents 2011; 37: 356–359. 10.1016/j.ijantimicag.2011.01.005 21353487

[23] Steward CD, Rasheed JK, Hubert SK, Biddle JW, Raney PM, Anderson GJ, et al Characterization of clinical isolates of *Klebsiella pneumoniae* from 19 laboratories using the National Committee for Clinical Laboratory Standards extended-spectrum β-lactamase detection methods. J Clin Microbiol. 2001; 39: 2864–2872. 10.1128/JCM.39.8.2864-2872.2001 11474005 PMC88252

[24] Pérez-Pérez FJ, Hanson ND. Detection of plasmid-mediated AmpC β-lactamase genes in clinical isolates by using multiplex-PCR. J Clin Microbiol. 2002; 40: 2153–2162. 10.1128/JCM.40.6.2153-2162.2002 12037080 PMC130804

[25] Yan JJ, Ko WC, Jung YC, Chuang CL, Wu JJ. Emergence of *Klebsiella pneumoniae* isolates producing inducible DHA-1 β-lactamase in a university hospital in Taiwan. J Clin Microbiol. 2002; 40: 3121–3126. 10.1128/JCM.40.9.3121-3126.2002 12202541 PMC130748

[26] Kim J, Lim YM, Rheem I, Lee Y, Lee JC, Seol SY, et al CTX-M and SHV-12 β-lactamases are the most common extended-spcetrum enzymes in clinical isolates of *Escherichia coli* and *Klebsiella pneumoniae* collected from 3 university hospitals within Korea. FEMS Microbiol Lett. 2005; 245: 93–98. 10.1016/j.femsle.2005.02.029 15796985

[27] Xu L, Ensor V, Gossain S, Nye K, Hawkey P. Rapid and simple detection of *bla*_CTX-M_ genes by multiplex PCR assay. J Med Microbiol. 2007; 54: 1183–1187. 10.1099/jmm.0.46160-0 16278432

[28] Ciesielczuk H, Hornsey M, Choi V, Woodford N, Wareham DW. Development and evaluation of a multiplex PCR for eight plasmid-mediated quinolone-resistance determinants. J Med Microbiol. 2013; 62: 1823–1827. 10.1099/jmm.0.064428-0 24000223

[29] Miyoshi-Akiyama T, Hayakawa K, Ohmagari N, Shimojima M, Kirikae T. Multilocus sequence typing (MLST) for characterization of *Enterobacter cloacae*. PLoS One 2013; 8: e66358 10.1371/journal.pone.0066358 23776664 PMC3679064

[30] Herschleb J, Ananiev G, Schwartz DC. Pulsed-field gel electrophoresis. Nat Protoc. 2007; 2: 677–684. 10.1038/nprot.2007.94 17406630

[31] Harada K, Shimizu T, Mukai Y, Kuwajima K, Sato T, Usui M, et al Phenotypic and molecular characterization of antimicrobial resistance in *Klebsiella* spp. isolates from companion animals in Japan: Clonal dissemination of multidrug-resistant extended-spectrum β-lactamase-producing *Klebsiella pneumoniae*. Front Microbiol. 2016; 7: 1021 10.3389/fmicb.2016.01021 27446056 PMC4925667

[32] Harada K, Niina A, Shimizu T, Mukai Y, Kuwajima K, Miyamoto T, et al Phenotypic and molecular characterization of antimicrobial resistance in *Proteus mirabilis* isolates from dogs. J Med Microbiol. 2014; 63, 1561–1567. 10.1099/jmm.0.081539-0 25187600

[33] Yamaguchi K, Ohno A, Ishii Y, Tateda K, Iwata M, Akizawa K, et al In vitro susceptibilities to levofloxacin and various antibacterial agents of 12,866 clinical isolates obtained from 72 centers in 2010. Jpn J Antibiotic. 2012; 65: 181–206. 23173294

[34] Harada K, Niina A, Nakai Y, Kataoka Y, Takahashi T. Prevalence of antimicrobial resistance in relation to virulence genes and phylogenetic origins among urogenital *Escherichia coli* isolates from dogs and cats in Japan. Am J Vet Res. 2012; 73: 409–417. 10.2460/ajvr.73.3.409 22369535

[35] Kanamori H, Yano H, Hirakata Y, Hirotani A, Arai K, Endo S, et al Molecular characteristics of extended-spectrum beta-lactamases and *qnr* determinants in *Enterobacter species* from Japan. PLoS One 2012; 7: e37967 10.1371/journal.pone.0037967 22719857 PMC3376121

[36] Mohd Khari Fl, Karunakaran R, Rosli R, Tee Tay S. Genotypic and phenotypic detection of AmpC β-lactamases in Enterobacter spp. isolated from a teaching hospital in Malaysia. PLoS One 2016; 11: e0150643 10.1371/journal.pone.0150643 26963619 PMC4786217

[37] Conceição T, Faria N, Lito L, Melo Cristino J, Salgado MJ, Duarte A. Diversity of chromosomal AmpC β-lactamases from *Enterobacter cloacae* isolates in a Portuguese hospital. FEMS Microbiol Lett. 2004; 230: 197–202. 14757240 10.1016/S0378-1097(03)00891-7

[38] Sheng WH, Badal RE, Hsueh RR; SMART Program. Distribution of extended-spectrum β-lactamases, AmpC-β-lactamases, and carbapenemases among *Enterobacteriaceae* isolates causing intra-abdominal infections in the Asia-Pacific region: results of the study for Monitoring Antimicrobial Resistance Trends (SMART). Antimicrob Agents Chemother. 2013; 57: 2981–2988. 10.1128/AAC.00971-12 23587958 PMC3697370

[39] Souna D, Amir AS, Bekhoucha SN, Berrazeq M, Drissi M. Molecular typing and characterization of TEM, SHV, CTX-M, and CMY-2 β-lactamases in *Enterobacter cloacae* strains isolated in patients and their hospital environment in the west of Algeria. Med Mal Infect. 2014; 44: 146–152. 10.1016/j.medmal.2014.01.008 24731757

[40] Harada K, Arima S, Niina A, Kataoka Y, Takahashi T. Characterization of *Pseudomonas aeruginosa* isolates from dogs and cats in Japan: current status of antimicrobial resistance and prevailing resistance mechanisms. Microbiol Immunol. 2012; 56, 123–127. 10.1111/j.1348-0421.2011.00416.x 22188523

[41] Harada K, Nakai Y, Kataoka Y. Mechanisms of resistance to cephalosporin and emergence of O25b-ST131 clone harboring CTX-M-27 β-lactamase in extraintestinal pathogenic *Escherichia coli* from dogs and cats in Japan. Microbiol Immunol. 2012; 56, 480–485. 10.1111/j.1348-0421.2012.00463.x 22486529

[42] Hayakawa K, Miyoshi-Akiyama T, Kirikae T, Nagamatsu M, Shimada K, Mezaki K, et al Molecular and epidemiological characterization of IMP-type metallo β-lactamase-producing *Enterobacter cloacae* in a large tertiary care hospital in Japan. Antimicrob Agents Chemother. 2014; 58: 3441–3450. 10.1128/AAC.02652-13 24709261 PMC4068452

[43] Kim ES, Jeong JY, Jun JB, Choi SH, Lee SO, Kim MN, et al Prevalence of *aac(6’)-Ib-cr* encoding a ciprofloxacin-modifying enzyme among *Enterobacteriaceae* blood isolates in Korea. Antimicrob Agents Chemother. 2009; 53: 2643–2645. 10.1128/AAC.01534-08 19289526 PMC2687187

[44] Huang S, Dai W, Sun S, Zhang X, Zhang L. Prevalence of plasmid-mediated quinolone resistance and aminoglycoside resistance determinants among carbapeneme non-susceptible *Enterobacter cloacae*. PLoS One 2012; 7: e47636 10.1371/journal.pone.0047636 23110085 PMC3479141

[45] Dalben M, Varkulja G, Basso M, Krebs VL, Gibelli MA, van der Heijden I, et al Investigation of an outbreak of *Enterobacter cloacae* in a neonatal unit and review of the literature. J Hosp Infect. 2008; 70: 7–14. 10.1016/j.jhin.2008.05.003 18632183

